# High prevalence and heterogeneity of Dysglycemia in patients with tuberculosis from Peru: a prospective cohort study

**DOI:** 10.1186/s12879-019-4416-2

**Published:** 2019-09-11

**Authors:** Roger I. Calderon, Maria B. Arriaga, Kattya Lopez, Nadia N. Barreda, Oswaldo M. Sanabria, José F. Fróes Neto, Davi Neri Araújo, Leonid Lecca, Bruno B. Andrade

**Affiliations:** 1 Socios En Salud Sucursal Peru, 15001 Lima, Peru; 2 Faculdade de Medicina, Universidade Federal do Rio de Janeiro, Rio de Janeiro, 21941-590 Brazil; 3 Faculdade de Medicina, Universidade Federal da Bahia, Salvador, Bahia 40110-100 Brazil; 4 Instituto Brasileiro para Investigação da Tuberculose, Fundação José Silveira, Salvador, Bahia 40210-320 Brazil; 5 Instituto Gonçalo Moniz, Fundação Oswaldo Cruz, Salvador, Bahia 40269-710 Brazil; 6 Multinational Organization Network Sponsoring Translational and Epidemiological Research (MONSTER) Initiative, Fundação José Silveira, Salvador, Bahia 40210-320 Brazil; 7 Curso de Medicina, Faculdade de Tecnologia e Ciências, Salvador, Bahia 41741-590 Brazil; 8 Department of Global Health and Social Medicine, Harvard Medical School, Boston, MA 02115 USA; 9 Universidade Salvador (UNIFACS), Laureate University, Salvador, Bahia 41720-200 Brazil; 10 Escola Bahiana de Medicina e Saúde Pública, Salvador, Bahia 40290-000 Brazil

**Keywords:** Diabetes mellitus, Prediabetes, Tuberculosis, Comorbidity, Prevalence

## Abstract

**Background:**

The accuracy of different laboratory tests for diagnosis of diabetes mellitus (DM) and prediabetes (preDM) in populations exposed to tuberculosis (TB) remains poorly understood. Here, we examined the prevalence of DM and preDM in TB affected people in Lima, Peru.

**Methods:**

A prospective cohort study of patients affected TB and their household contacts (HHC), was conducted between February and November 2017 in Lima, Peru. Fasting plasma glucose (FPG), HbA1c and oral glucose tolerance test (OGTT) were used to detect DM and preDM in a prospective cohort of TB patients (*n* = 136) and household contacts (*n* = 138). Diagnostic performance of the laboratory tests was analyzed. Potential effects of sociodemographic and clinical factors on detection of dysglycemia were analyzed.

**Results:**

In TB patients, prevalence of DM and preDM was 13.97 and 30.88% respectively. Lower prevalence of both DM (6.52%) and preDM (28.99%) were observed in contacts. FPG, HbA1c and OGTT had poor agreement in detection of preDM in either TB cases or contacts. TB-DM patients had substantially lower hemoglobin levels, which resulted in low accuracy of HbA1c-based diagnosis. Classic sociodemographic and clinical characteristics were not different between TB patients with or without dysglycemia.

**Conclusion:**

High prevalence of DM and preDM was found in both TB patients and contacts in Lima. Anemia was strongly associated with TB-DM, which directly affected the diagnostic performance of HbA1c in such population.

**Electronic supplementary material:**

The online version of this article (10.1186/s12879-019-4416-2) contains supplementary material, which is available to authorized users.

## Background

Tuberculosis kills 1–2 million people per year, especially in low and middle-income countries, and despite recent efforts to improve control programs, its incidence is declining at slow rates [1]. High-risk groups undermine the success of TB programs in the reducing the disease burden notwithstanding targeted interventions. One relevant aspect is diabetes mellitus (DM), which increases the risk of developing active TB in approximately three times [2]. More recently, prediabetes (preDM) has been also described as a risk factor for developing TB [3, 4]. Both the World Health Organization (WHO) as the Peruvian National TB Program (NTP) recommend screening for DM in people with active TB and for TB between household contacts [5]. Despite those indications, most individuals are unaware of their DM or pre-DM status. In 2017, the Peruvian NTP communicated a DM incidence of 6.2% with a testing coverage of 77.9% of all TB patients whereas other instances of the national government reported in 2016 a DM incidence around 10.4% (government communication). Those differences may reflect several limitations in the DM screening such as the use of only fasting plasma glucose (FPG) as the screening approach [5]. It is widely known that sensitivity of DM tests (such as HbA1c, fasting glucose and oral glucose tolerance) is variable [6–9]. It has been reported that HbA1c detects more people with DM [10] (or preDM) with higher sensitivity than other screening tests [11], so systematic use of FPG as screening tool, as observed in Peru, may result in incomplete record of TB-DM cases [12].

Due the discrepancies in sensitivity of DM screening tests and in order to determine the real prevalence of DM or preDM in TB patients and their household contact (HHC), we conducted a study in Lima, Peru, with the hypothesis that both DM and pre-DM are more frequent than previously officially reported. In addition, we compared the performance of different screening methods to detect DM and preDM in TB cases. Furthermore, we examined the associations between different other clinical and sociodemographic risk factors with the possible more extended comorbid TB-DM burden.

## Methods

### Study design

**Fig. 1:**
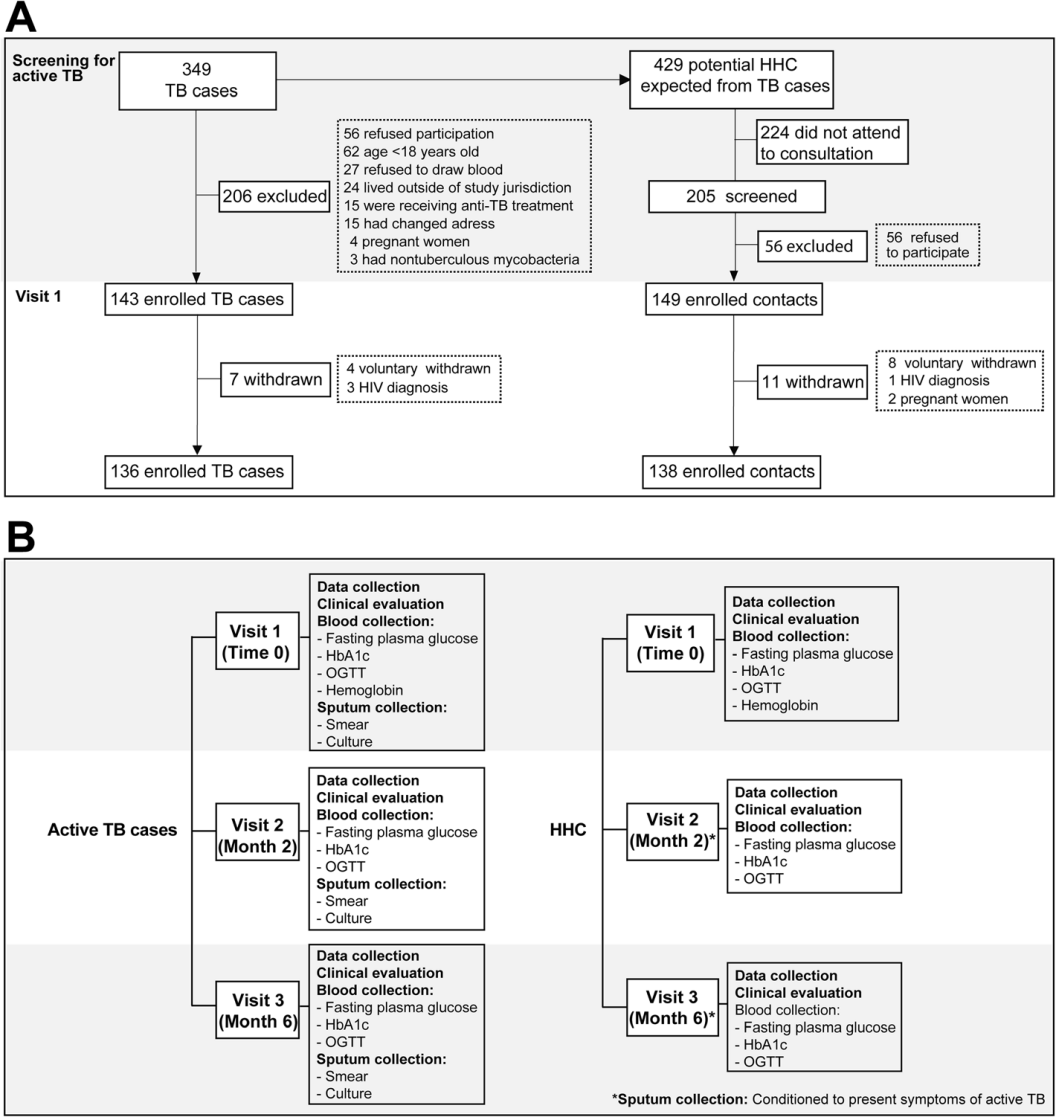
Study outline. **a** Flowchart describing the recruitment of the study population. **b** Flowchart of the investigations and procedures performed

This was a prospective cohort study of patients affected TB and their household contacts (HHC), conducted between February and November 2017 in Lima, Peru. The recruitment details, as well as the procedures and investigations performed are outlined in Fig. 1a-b.

### Study population

The study was carried out in the Public Hospital Sergio Bernales and outpatient health centers of Carabayllo and Comas districts. Patients with pulmonary TB with ≥16 years of age diagnosed by the NTP of public health centers, who are not receiving anti-TB treatment or have started it in a period of no more than 5 days, were included. In this study, HHC was defined as a person 12 years of age or older who shared at least household where they sleep or take their meals (at least one of them per day) with a study TB index patient. Exclusion criteria were patients or contacts diagnosed with HIV, pregnant women, who did not live permanently in the jurisdiction area of the study and patients who had infection or disease due to non-tuberculous mycobacteria. The follow-up of those patients was conducted up to 6 to 12 months after enrollment.

### Study power and sample size calculations

Power calculations were performed using the software OpenEpi 3.0 (www.openepi.com). We expected that 130 individuals per group (TB cases and HHC) would result in about 80% of power to detect difference in DM prevalence of 10% (14% in TB cases and 4% in HHC), at a significance level of α = 0.05.

### Laboratory and field procedures

One sputum specimen for each patient was isolated for smear investigation (acid-fast bacilli, AFB) and well as *Mycobacterium tuberculosis* (Mtb) solid cultures. For AFB investigation, sputum smears were stained with Ziehl–Neelsen and examined by microscopy. The remaining sputum samples were cultured for Mtb at baseline and different study visits until completion of anti-TB treatment. Positive smears of Mtb were graded as 1+ (10–99 AFB observed in 100 fields), 2+ (1 a 10 AFB in 50 fields) and 3+ (more than 10 AFB in 20 fields). For observations up to 9 AFB, exact number of AFB was recorded as previously described [13]. Mtb positive cultures were graded as 1+ (20–100 colonies), 2+ (more than 100 discrete colonies) and 3+ (more than 100 colonies forming a confluent mass). For growth of up to 19 colonies, exact number of colonies was recorded as previously described [14]. Mtb complex and drug susceptibility testing were performed following standard procedures reported elsewhere [15]. All the TB testing were performed at the Socios En Salud (SES) Laboratory located in Lima.

DM was defined in agreement with American Diabetes Association (ADA) guidelines [16] as 2-h glucose ≥200 mg/dL (OGTT), HbA1c ≥6.5% or fasting plasma glucose ≥126 mg/dL. PreDM was also defined in agreement with ADA guidelines as 2-h glucose 140 a 199 mg/dL, HbA1c 5.7 a 6.4% or fasting plasma glucose 100–125 mg/dL. The measurement of HbA1c in whole blood specimens was performed by use of TRI-stat™ platform (Trinity Biotech, Ireland) and fast serum glucose or glucose level on OGTT test, was performed following standard methods. Of note, OGTT was performed only in individuals without prior DM diagnosis following instructions from the Institutional Review Board which handled the Ethical aspects of the study protocol.

Anemia was defined following WHO criteria as hemoglobin (Hb) level below 12.5 g/dL for female and 13.5 g/dL for male. Hb measurement was performed in whole blood specimens stored at − 80 °C from patients and HHC around one year after the blood sample collection at the end of the enrollment. One 50 μL-aliquot of whole blood of each participant was thawed and separated in a new tube to be passed through the HumaCount 5D Hematology System (Wiesbaden, Germany).

The SES lab conducts annual external quality assurance through competition panels of the College of American Pathologists (Northfield, Illinois) and other agencies.

### Clinical data

Clinical evaluations were also carried out by a specialist, interviews for the collection of socio-demographic and clinical information, review of medical records to obtain relevant clinical information on comorbidities such as blood pressure, pulse, respiratory rate, immunosuppressive conditions, among others. Hypertension defined by WHO criteria: measured on two different days, the systolic blood pressure readings on both days is ≥140 mmHg and/or the diastolic blood pressure readings on both days is ≥90 mmHg. The anthropometric measurement was performed: Weight, height and abdominal circumference measurement. Demographic and clinical information were registered in the Socios En Salud Informatic System (SEIS) software (Lima, Peru).

### Outcomes

Treatment outcome was classified as successful for patients considered microbiologically “cured” or (negative Mtb cultures and AFB negative in sputum smears at the end of treatment), whereas “death”, “default” (treatment dropout/abandonment) and “failure” (positive cultures at the end of treatment) were combined as poor outcome [17]. The primary outcome in this study was the prevalence of DM and preDM in patients with TB and their HHC using the fasting serum glucose assays, HbA1c or OGTT (this one only in people without prior DM diagnosis), evaluated and indicated by the endocrinologist and also following the considerations of the American Diabetes Association.

### Data analysis

We assessed the diagnostic performance of FPG and HbA1c for DM and preDM using the conjunction of OGTT testing and clinical DM/preDM diagnosis, both as gold standard. Characteristics of study participants were presented as median and interquartile ranges (IQR) for continuous variables or frequency for categorical variables. Continuous variables were compared using the Mann-Whitney *U* test (between two groups) or Kruskal-Wallis test with Dunn’s multiple comparisons. Categorical variables were compared using the Fisher’s exact test (2 × 2 comparisons) or Pearson’s chi square test. We constructed Receiver Operating Characteristic (ROC) curves to calculate sensitivity, specificity, and predictive values determined at different cut-off values for HbA1c and FPG. Finally, the Kappa (K) statistic was calculated to assess agreement between OGTT versus FPG or HbA1c as diagnostic test for DM or preDM. The Kappa statistic was interpreted by Landis and Koch criteria [18]. All analyses were pre-specified. Two-sided *P* value < 0.05 after adjustment for multiple comparisons (Bonferroni’s method) were considered statistically significant. Statistical analyses were performed using SPSS 24.0 (IBM statistics), Graphpad Prism 7.0 (GraphPad Software, San Diego, CA) and JMP 13.0 (SAS, Cary, NC, USA).

## Results

We initially screened 349 microbiologically-confirmed TB cases at the primary health care centers which were part of the present study, between February and November 2017. During the screening, 206 individuals were excluded for a number of reasons listed in Fig. 1a, and 143 patients with active TB were examined in the first study visit (Fig. 1a and Fig. 1b). At this stage, additional 7 persons were excluded due to HIV diagnosis (*n* = 3) and consent withdraw (*n* = 4), resulting in a cohort of 136 patients. During the search of HHCs of the TB index cases, 205 people were identified for screening and only 149 were effectively screened. Of those, 8 withdrew consent and 3 were excluded for having positive HIV status (*n* = 1) or pregnancy (*n* = 2) resulting in a total of 138 HHC participants (Fig. 1).

**Fig. 2:**
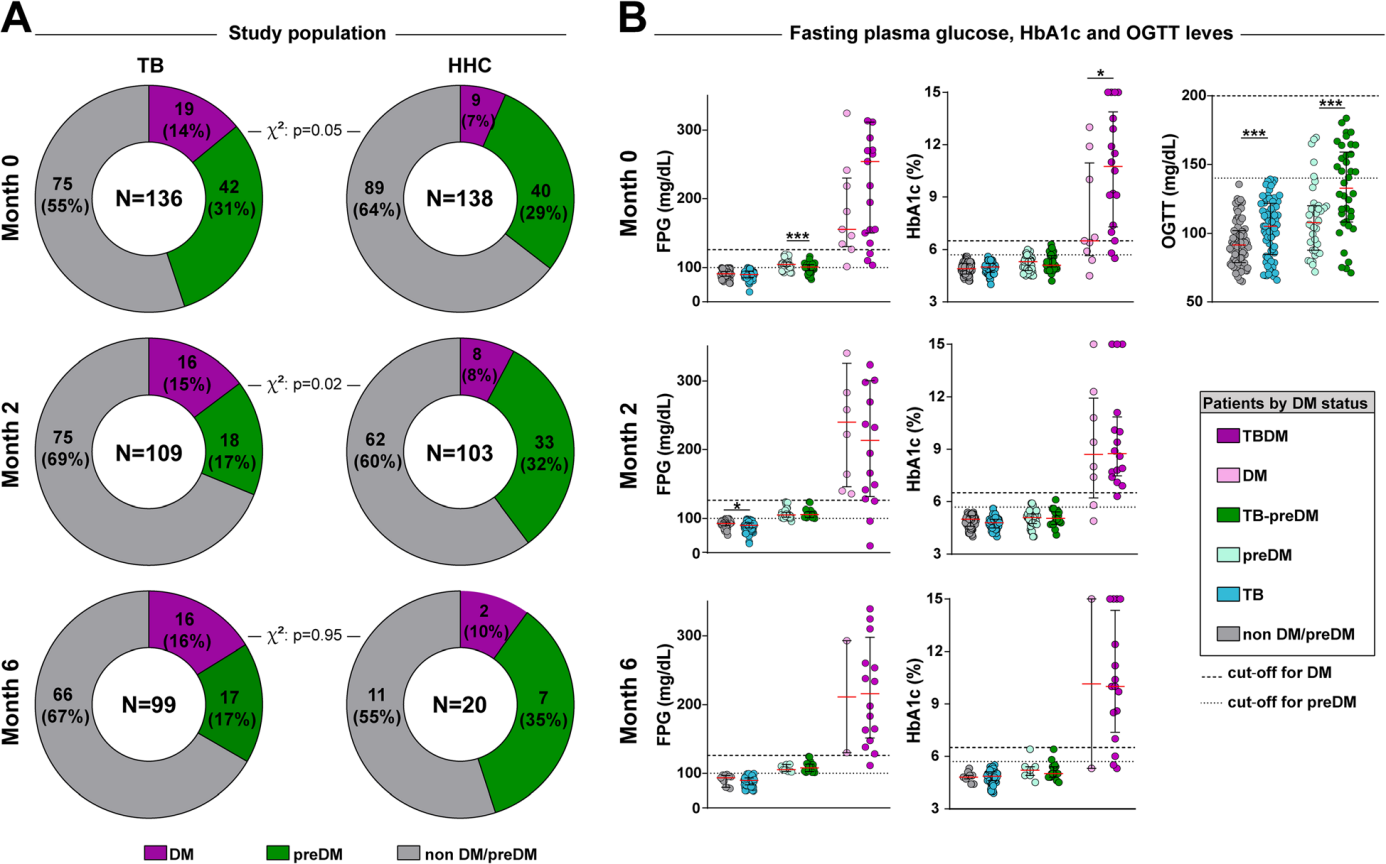
Prevalence of DM and preDM in TB patients and HHC at different study timepoint. **a** Prevalence of DM and preDM between TB patients and HHC. **b** Scatter plots depicting the distribution of serum glucose, HbA1c and OGTT values in TB patients and household contacts. Lines represent median and interquartile range values at different study timepoints. HHC: Household contact; FPG: Fasting Plasma Glucose; HbA1c: Glycated Hemoglobin; OGTT: Oral Glucose Tolerance Test. DM and preDM categories into TB and HHC groups were compared using Chi-square test (**a**). The differences in median values (and IQR) of Glucose, HbA1c and OGGT between DM and preDM (into TB cases and HHC) groups were compared using the Mann-Whitney *U* test (**b**). Only comparisons with significant p-values are displayed (*p < 0.05, **p < 0.01, ***p < 0.001)

At enrollment, the DM prevalence was 13.97% (95% CI: 8.14, 19.80%) (*n* = 19) among TB patients (all of whom refereed DM diagnosed prior to study enrollment), while the prevalence of DM among HHC was 6.52% (95% CI: 2.40, 10.64%) (*n* = 9). The prevalence of preDM was 30.88% (95% CI: 23.81, 38.65%) in TB patients (*n* = 42) and 28.99% (95% CI: 21.42, 36.56%) among the HHC (*n* = 40). Diagnosis of preDM was performed at study enrollment. Comparisons at this timepoint revealed that TB and HHC groups exhibited similar frequencies of DM and preDM (Fig. 2a). After 2 months of antitubercular treatment (ATT) commencement, DM frequency was nearly double in TB vs. HHC whereas preDM was higher in the latter group (Fig. 2a). At month 6 of ATT, frequency of DM and preDM was once again not different between the study groups, although there was a remarkable 75% loss to follow-up in the HHC group at this timepoint (Fig. 2a).

Among the TB index cases at study baseline, DM individuals were on average older than preDM and normoglycemic individuals (median age: 46.41 yrs. IQR:33.5–54.8 vs. 39.8 yrs., IQR: 26.7–54.0 and 26.4 yrs. IQR: 22.3–34.7, respectively, *p* < 0.01) (Table 1). In addition, lower level of education (primary and secondary school years) was associated with presence of dysglycemia (DM or preDM) (*p* < 0.01; Table 1). Hypertension was more frequently in DM patients (21.1%) than that in normoglycemic TB cases (7.3%) (*p* < 0.01). The study groups were similar with regard to a number of other characteristics including sex, history of prior TB, asthma, renal disease and lifestyle habits (alcohol use, smoking and illicit drugs use) (Table 1).

**Table 1:** Characteristics of pulmonary TB cases stratified according to glycemic status

Characteristics	n/N	TBDM	TBpreDM	Normoglycemic	*p*-value
		*n* = 19	*n* = 42	*n* = 75	
Age (years)-median (IQR)	136/136	46.41 (33.5–54.8)	39.8 (26.7–54.0)	26.4 (22.3–34.7)	**< 0.01**
Gender	136/136				0.354
Male	136/136	9 (47.4)	27 (64.3)	47 (62.7)	
Female	136/136	10 (52.6)	15 (35.7)	28 (37.3)	
Prior TB	136/136	5 (26.3)	8 (19.0)	10 (13.3)	0.158
BCG vaccination	135/136	17 (89.5)	38 (92.7)	71 (94.7)	0.411
Smoking	135/136	4 (21.1)	10 (24.4)	15 (20.0)	0.767
Smokers at home	135/136	2 (10.5)	4 (9.8)	5 (6.7)	0.499
Cannabis use	135/136	1 (5.3)	7 (17.1)	13 (17.3)	0.283
Illicit drug use	135/136	2 (10.5)	7 (17.1)	8 (10.7)	0.707
Alcohol use	135/136	4 (21.1)	29 (70.7)	37 (49.3)	0.348
Hypertension	135/136	4 (21.1)	3 (7.3)	0 (0.0)	**< 0.01**
Asthma	135/136	0 (0.0)	3 (7.3)	5 (6.7)	0.399
Renal disease	135/136	1 (5.3)	0 (0.0)	2 (2.7)	0.844
Slow scarring	135/136	3 (15.8)	9 (22.0)	7 (9.3)	0.186
Metformin use	132/136	7 (36.8)	0 (0.0)	0 (0.0)	**< 0.01**
BMI (kg/m2)-median (IQR)	132/136	22.3 (21.4–26.4)	23.2 (21.2–25.1)	22.3 (20.3–25.4)	0.749
Waist circumference (cm) -median (IQR)	132/136	85 (81–89)	88 (80–91)	83 (76–88)	0.058
Hb (g/dL) -median (IQR)	134/136	9.9 (7.6–13.4)	12.0 (10.8–13.1)	12.6 (11.3–13.4)	
Anemia	136/136	19 (100)	31 (73.8)	49 (66.2)	**0.006**
FPG (mg /dL) -median (IQR)	136/136	218.9 (147.7–298.1)	103.1 (100.4–106.3)	89.7 (85.6–93.9)	**< 0.01**
HbA1c (%)-median (IQR)	134/136	10.8 (7.4–13.5)	5.1 (4.8–5.5)	5.00 (4.6–5.1)	**< 0.01**
OGTT (mg/dL) -median (IQR)	112/136	119.5 (119.5–119.5)	128.5 (106.5–157.1)	105.2 (85.8–121.4)	**< 0.01**
AFB smear	135/136				**0.004**
Negative		5 (26.3)	15 (35.7)	40 (54.1)	
1+		3 (15.8)	9 (21.4)	15 (20.3)	
2+		3 (15.8)	6 (14.3)	6 (8.1)	
3+		7 (36.8)	10 (23.8)	11 (14.9)	
Scanty		1 (5.3)	2 (4.8)	2 (2.7)	
L-J culture	131/136				0.59
Negative		4 (21.1)	9 (22.5)	25 (34.2)	
1+		12 (63.2)	18 (45.0)	33 (45.2)	
2+		2 (10.5)	4 (10.0)	3 (4.2)	
3+		0 (0.0)	4 (10.0)	3 (4.2)	
colonies		1 (5.3)	5 (12.5)	8 (11.1)	
BD MGIT™ 960 System	78/136				0.11
Positive		8 (80.0)	22 (81.5)	34 (82.9)	
Negative		2 (20.7)	5 (18.5)	7 (17.1)	
MDR	76/136	2 (18.2)	3 (12.5)	4 (9.8)	0.451
Isoniazid-resistant	76/136	2 (18.2)	5 (20.8)	7 (17.1)	0.831
Rifampicin-resistant	76/136	2 (18.2)	4 (16.7)	5 (12.2)	0.55
TB treatment outcome	115/136				0.64
Poor		5 (27.8)	8 (23.5)	14 (22.2)	
Cure		13 (72.2)	26 (76.5)	49 (77.8)	
Polyuria	136/136	9 (47.4)	16 (38.1)	28 (37.3)	0.493
Polydipsia	136/136	9 (47.4)	20 (47.6)	36 (48.0)	0.956

Data represent no. (%); IQR: Interquartile range. *BCG* Bacillus Calmette–Guérin, *BMI* Body Mass Index, *Hb* Hemoglobin, *FPG* Fasting Plasma Glucose, *HbA1c* Glycated Hemoglobin, *OGTT* Oral Glucose Tolerance Test, *AFB* Acid-Fast Bacilli, *L-J* Löwenstein-Jensen, *MDR* Multi Drug Resistant. Hypertension, asthma, renal disease and anemia as defined by the World Health Organization as described in Methods. Prior TB: diagnosis of active tuberculosis before of this

When the cohort of HHC was stratified according to the glycemic status, we found once again that DM patients were on average older than those with preDM or normoglycemia (Table 2). In addition, the highest values of body mass index (BMI) and waist circumference were detected in the subgroup of preDM HHC (Table 2). Other characteristics were similar between the HHC subgroups.

**Table 2:** Characteristics of household contacts of pulmonary TB cases stratified glycemic status

Characteristics	n/N	DM	preDM	Normoglycemic	*p*-value
		*n* = 9	*n* = 40	*n* = 89	
Age (years) -median (IQR)	138/138	60.9 (57.5–68.0)	49.2 (40.0–57.3)	30.45 (65.83)	**< 0.01**
Gender	136/138				0.44
Male		5 (55.6)	21 (52.5)	30 (34.5)	
Female		4 (44.4)	19 (47.5)	57 (65.5)	
Education	138/138				0.207
Primary or secondary		9 (100.0)	33 (82.5)	71 (79.8)	
Technical or university		0 (0.0)	7 (17.5)	18 (20.2)	
Prior TB	135/138	2 (22.2)	3 (7.7)	9 (10.3)	0.618
BCG vaccination	135/138	9 (100.0)	36 (92.3)	84 (96.6)	0.752
Smoking	135/138	0 (0.0)	7 (17.9)	12 (13.8)	0.682
Smokers at home	135/138	2 (22.2)	6 (15.4)	11 (12.6)	0.427
Cannabis use	134/138	0 (0.0)	2 (5.1)	0 (0.0)	0.185
Illicit drug use	134/138	0 (0.0)	1 (2.6)	0 (0.0)	0.35
Alcohol use	134/138	2 (22.2)	22 (56.4)	25 (29.1)	0.134
Hypertension	135/138	0 (0.0)	6 (15.4)	6 (6.9)	0.647
Asthma	135/138	0 (0.0)	3 (7.7)	7 (8.0)	0.515
Renal disease	135/138	0 (0.0)	3 (7.7)	4 (4.6)	0.978
Slow scarring	135/138	2 (22.2)	3 (7.7)	6 (6.9)	0.229
Metformin use	133/138	2 (22.2)	0 (0.0)	0 (0.0)	**< 0.01**
Consanguinity with index case	135/138	3 (33.3)	3 (7.7)	15 (17.2)	0.96
BMI (kg/m2) -median (IQR)	133/138	29.41 (26.9–31.7)	29.8 (28.1–33.4)	26.1 (2.9–29.4)	**< 0.01**
Waist circumference (cm) -median (IQR)	133/138	95 (93–105)	98 (94–107)	88 (79–94)	**< 0.01**
Hb (g/dL) -median (IQR)	138/138	13.2 (12.9–13.7)	13.6 (12.8–14.6)	13.1 (12.1–14.2)	0.151
Anemia	138/138	2 (22.2)	8 (20.0)	35 (39.3)	0.138
FPG (mg /dL) -median (IQR)	138/138	155.60 (134.3–218.3)	104.6 (102.0–107.9)	90.9 (87.5–94.2)	**< 0.01**
HbA1c (%)-median (IQR)	138/138	6.5 (5.9–10.0)	5.3 (4.8–5.50)	4.9 (4.6–5.2)	**< 0.01**
OGTT (mg/dL) -median (IQR)	124/138	91.6 (70.3–107.10)	107.8 (87.7–119.8)	91.6 (78.9–101.9)	**< 0.01**
Polyuria	138/138	5 (55.6)	7 (17.5)	19 (21.3)	0.187
Polydipsia	138/138	3 (33.3)	14 (35.0)	19 (21.3)	0.124
Malaise	138/138	2 (22.2)	10 (25.0)	24 (27.0)	0.721

Data represent no. (%); *IQR* Interquartile range, *BCG* Bacillus Calmette–Guérin, *BMI* Body Mass Index, *Hb* Hemoglobin, *FPG* Fasting Plasma Glucose, *HbA1c* Glycated Hemoglobin, *OGTT* Oral Glucose Tolerance Test. Hypertension, asthma, renal disease and anemia as defined by the World Health Organization as described in Methods

The FPG and HbA1c tests were performed prospectively in all patients recruited (except OGTT for known DM cases). At the baseline visit, as expected, TB patients with coincident DM exhibited higher values of FPG and HbA1c than those diabetic but without TB (Fig. 2b). Such discrepancies in laboratory measurements were reduced at month 2 and 6 of ATT and no statistically significant differences were observed between diabetic patients with or without TB (Fig. 2b). The OGTT was performed only at baseline in patients without prior DM diagnosis. One individual was identified as having DM (from the HHC group). Once again, presence of TB was associated with higher values of OGTT results than in HHC individuals, suggesting that Mtb infection may drive inflammation-associated dysglycemia [19, 20] (Fig. 2b).

**Fig. 3:**
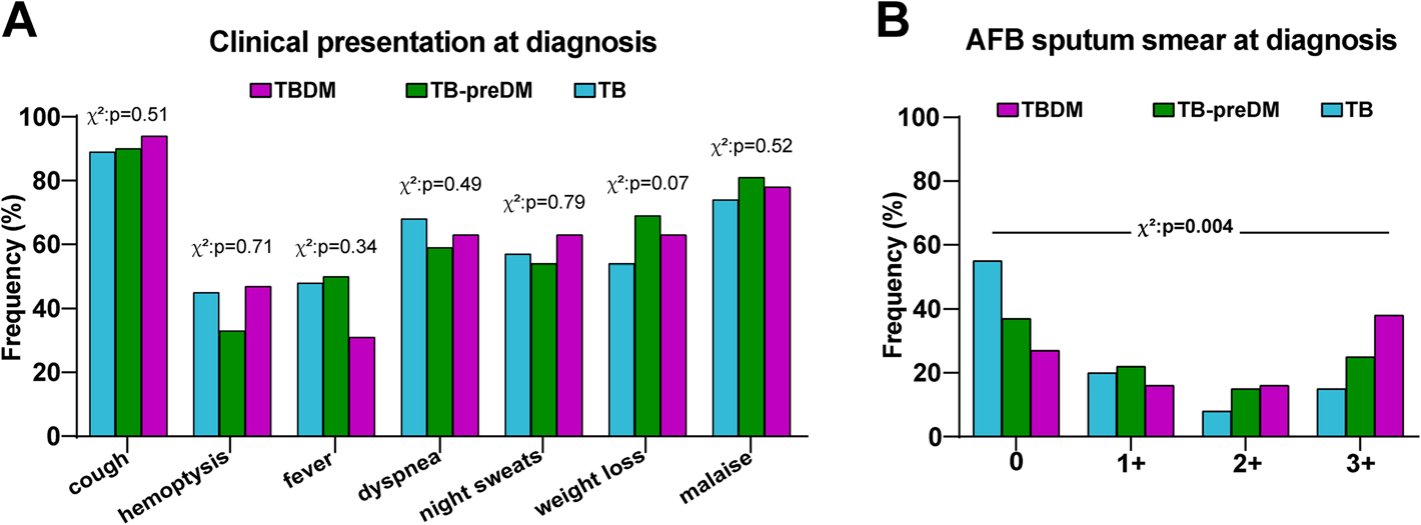
TB-diabetes comorbidity is associated with increased mycobacterial loads in sputum smears, but in frequency of TB-related symptoms. **a** Prevalence of indicated TB-related symptoms was compared between TB patients with diabetes (TBDM), prediabetes or normoglycemia. **b** Frequency of TB patients with different AFB smear grades in sputum classified according to glycemic status as indicated. Data were analyzed using the Pearson’s chi-square test

Moreover, the frequencies of TB-related symptoms between TB index cases with different degree of dysglycemia were compared and we found no statistically significant difference (Fig. 3a). Moreover, frequency patients presenting with increased acid-fast bacilli grades in sputum smears was higher in TB cases with DM compared to the other groups (chi-square *p* = 0.004; Fig. 3b). These findings indicate that TB clinical presentation is not worse in diabetics as we have previously demonstrated in different studies from Brazil [3, 19], but confirmed previously reported data showing increased mycobacterial loads in sputum smears [19].

**Fig. 4:**
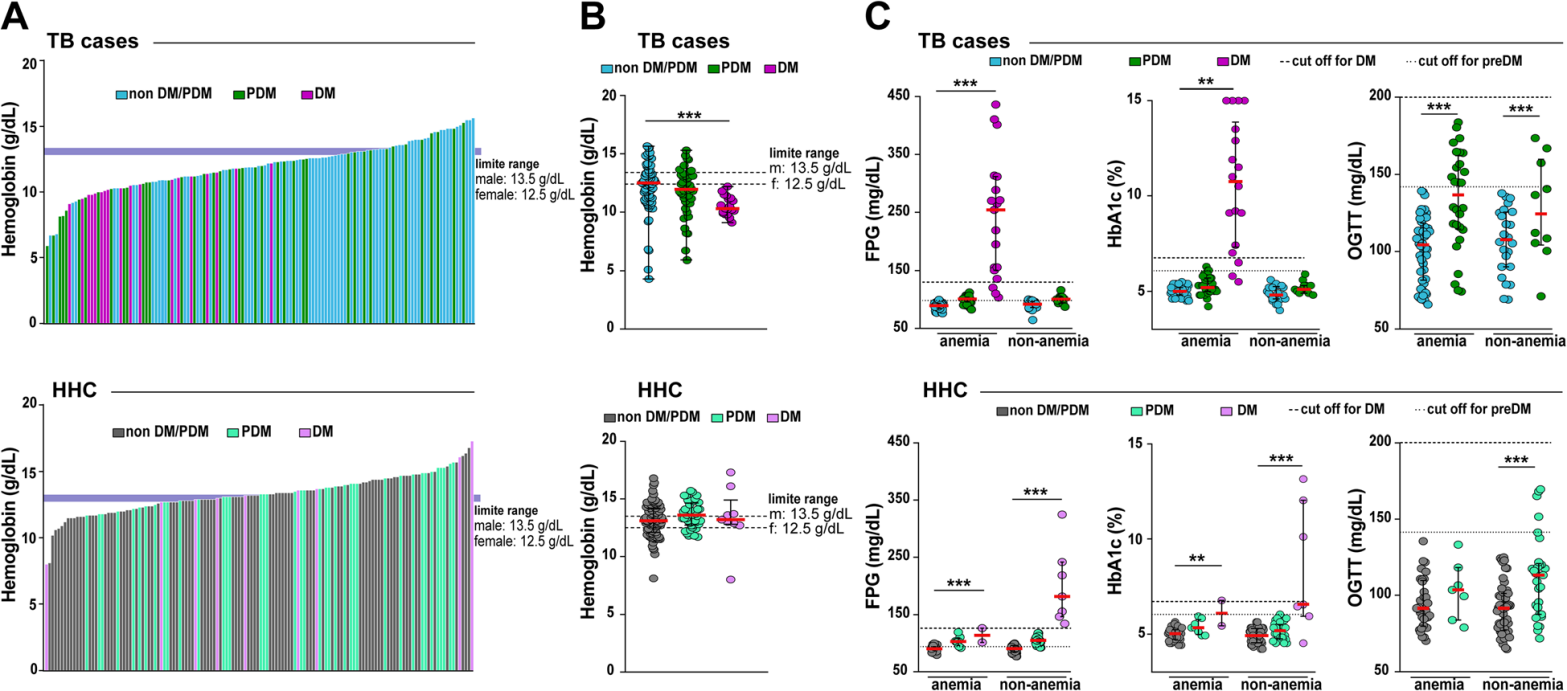
Impact of anemia on the tests used for screening of dysglycemia in the study population. **a** Histograms representing hemoglobin levels in TB or HHC are shown. Scatter plots depicting the distribution of hemoglobin values (**b**) as well as of levels of PFG, HbA1c or OGTT (**c**) in individuals from TB or HHC study groups stratified by anemia status are shown. HbA1c: Glycated Hemoglobin; OGTT: Oral Glucose Tolerance Test. The differences in median values (and IQR) of Glucose, HbA1c and OGGT between DM and preDM (into TB cases and HHC) groups were compared using the Kruskal-Wallis test with Dunn’s multiple comparisons (B-C). Only comparisons with significant *p*-values are displayed (*p < 0.05, **p < 0.01, ***p < 0.001)

Circulating hemoglobin levels were low in diabetic patients with coincident TB compared to those with preDM or normoglycemia (Fig. 4a and b). In fact, within the group of TB, anemia was detected in all diabetics but only in 73.8% of preDM (*n* = 31) and 66.2% of normoglycemic patients (*n* = 49) (*p* = 0.006). In HHC, diabetes was not associated with substantial changes in hemoglobin levels (Fig. 4a and b). Moreover, frequency of anemia was not different between the groups of TB contacts presenting with diverse degree of dysglycemia (*p* = 0.08). This finding suggested that a potential synergistic effect of DM and TB on the degree of anemia may exist. Indeed, results from FPG and HbA1c in TB patients demonstrated that values observed in DM were substantially higher than in preDM or normoglycemia, and similar trend was observed in OGTT between preDM and nondiabetics (Fig. 4c). Of note, in HHC, values of FPG and HbA1c were lower in anemic individuals compared to those with normal hemoglobin levels (Fig. 4c). Furthermore, OGTT levels were able to distinguish preDM from normoglycemia only in the subgroup of participants without anemia (Fig. 4c).

**Fig. 5:**
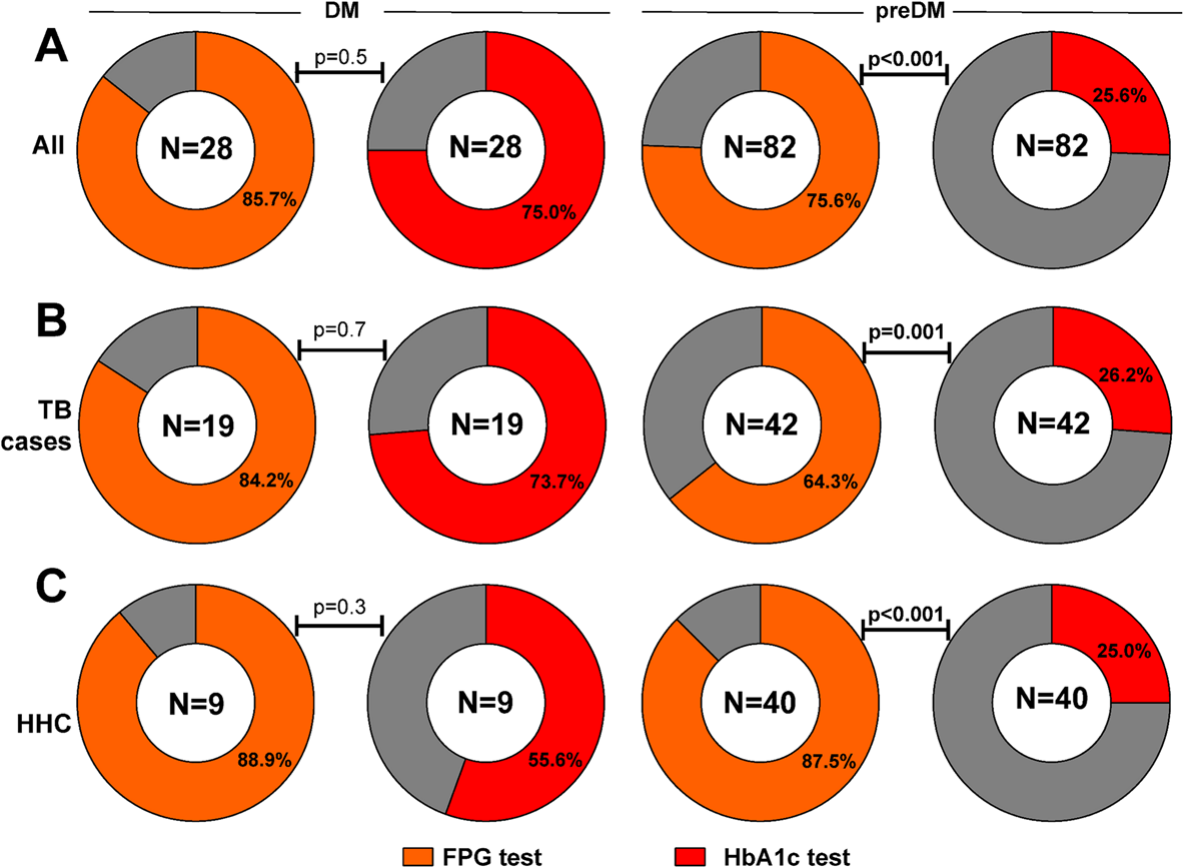
Detection of diabetes and prediabetes using FPG or HbA1c. Frequency of individuals with diabetes (DM) or prediabetes (PDM) correctly diagnosed using either fasting plasma glucose (FPG) or HbA1c levels is shown for all participants (**a**) as well as for only TB patients (**b**) or household contacts (HHC) (**c**). Frequency of detection of DM or PDM between FPG and HbA1c was compared using the Fisher’s exact test

**Fig. 6:**
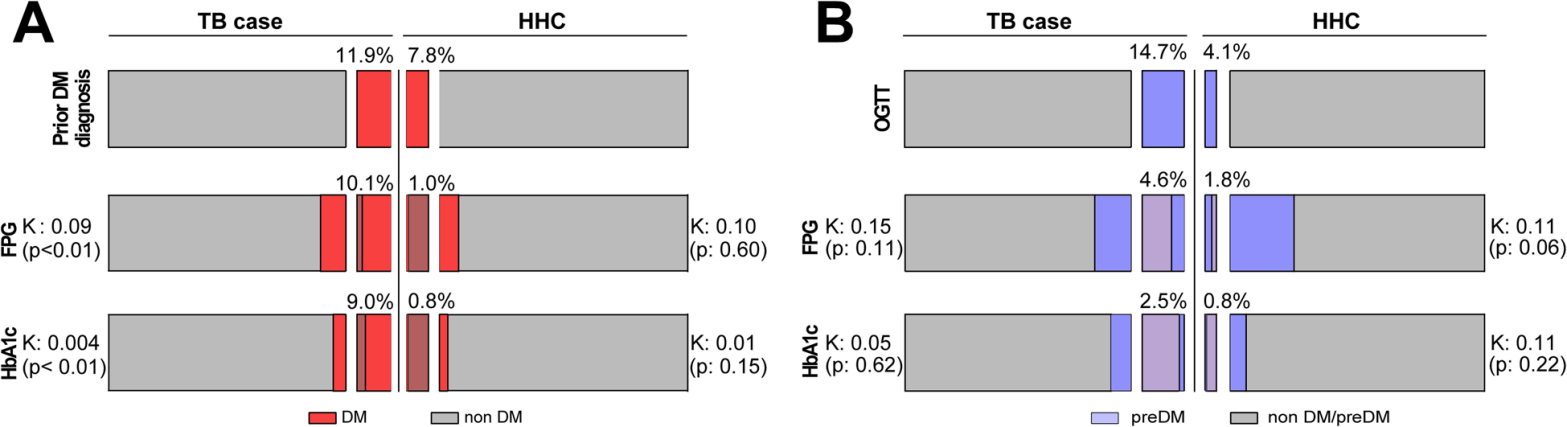
Concordance Kappa (K) analysis between HbA1c, FPG and OGTT to detect diabetes or prediabetes in TB cases and HHC. **a** Results for TB cases (reference standard was prior DM diagnosis). **b** Results for HHC (reference value was OGGT). PD: Prior Diabetes Mellitus diagnosis; FPG: Fasting Plasma Glucose; HbA1c: Glycated Hemoglobin; OGTT: Oral Glucose Tolerance Test

We next analyzed the overall accuracy of FPG and HbA1c in detecting DM or preDM cases in individuals stratified according to TB diagnosis (TB patients and HHC). FPG levels were able to detect more cases with preDM, but not with DM, than HbA1c did in the entire study population (Fig. 5a). When only TB cases were considered, again FPG was able to detect more preDM patients then HbA1c (Fig. 5b). Similar findings were observed in HHC (Fig. 5c). These results indicate that the laboratory tests examined here displayed different values of accuracy to detect DM or preDM in the study population. Summary of the accuracy and predictive values are shown in Additional file 1: Table S1. Concordance on DM diagnosis using either FPG or HbA1c levels was low, with lower performance of HbA1c (Fig. 6a). In addition, poor agreement was also observed between OGTT versus HbA1c or FPG tests to detect preDM in both TB patients and HHC (Fig. 6b).

## Discussion

Estimating the prevalence of dysglycemia among TB cases is important to understand the real burden of TBDM and drive changes to optimize detection and treatment of this comorbidity. In the present study, high prevalence of DM and preDM was found in both TB patients (14%) and HHC (6.5%), which is unexpectedly higher than previous reports from the Peruvian Minister of Health (6.2%) [21], but similar to results from a recent report [22]. In the last years, low and middle income countries, such as Peru, have experienced a nutritional transition [23]. These populations historically affected by hunger and malnutrition now face additional problems of obesity and other non-communicable diseases, such as diabetes and hypertension [24]. Because DM is a known risk factor of TB [2, 25, 26], systematic screening should be performed in high-risk populations. Nevertheless, maybe due to high cost, many countries do not perform systematic screening and for this reason the dual burden of TB and DM is likely still underestimated. Findings from this study and others [27, 28] argue that, to provide a more reliable picture of the TBDM/preDM burden and based on some other references, it is necessary to consider the joint use of FPG and HbA1c in all patients, while OGTT could be used, when it is safe, to rule out prediabetes.

In our prospective evaluation, we found that frequency of DM remained stable during the follow up period, including in the group of TB patients. This could be due to the fact that most of the DM patients investigated exhibited high levels of HbA1c, compatible with uncontrolled diabetes [29] and these values remained high during the follow-up, in disagreement to results from a previous report [30]. We hypothesize that patients with very high levels of HbA1c are not able to substantially decrease values after ATT-induced reduction of systemic inflammation. On the other hand, frequency of preDM significantly reduced in TB patients undergoing ATT but were again stable in the follow up timepoint of the HHC cohort. This observation argues that resolution of systemic inflammation during ATT may lead to reduction of transient dysglycemia detected in some patients with TB.

There is evidence that TB clinical presentation is exacerbated in individuals [25, 31, 32]. In addition, poor glycemic control has been described to negatively impact radiographic manifestation of pulmonary disease in patients with TBDM [25]. In agreement with this idea, we have recently described in two distinct studies from Brazil that DM and also preDM were associated with increased frequency of clinical symptoms associated with TB [19]. Nevertheless, the association between DM and worse TB clinical and/or radiographic presentation has not been found by other investigations, including a recent study which examined Peruvian individuals [33]. It is possible that genetic or social differences between countries may influence the perception of the symptoms. Moreover, differences in timing from disease onset to admission at a TB clinical center may diminish the differences in clinical symptoms between normoglycemic and dysglycemic patients. Further studies are warranted to directly test these hypotheses. Interestingly, we found that TBDM patients exhibited increased AFB grades in sputum smears, which has been described by independent investigations worldwide [3, 19]. These findings reinforce the idea that DM may impact capability to restrain mycobacterial growth and thus may be associated with increased risk of transmission.

Anemia is a common clinical condition associated with TB [34]. It has been recently reported that chronic anemia is linked to a distinct systemic inflammatory profile that persists after 2 months of ATT in a Brazilian cohort [26]. In addition, hemoglobin levels have been described to affect detection of glycated hemoglobin, which brings potential challenges in DM diagnosis in patients with severe anemia [26]. Herein, all the TB-DM patients were anemic (median Hb level: 9.9 g/dL IQR: 7.6–13.4 g/dL). However, anemic status apparently did not prevent detection of DM in TB patients as the screening tests exhibited similar performance. Whether anemia impacts TB clinical presentation in DM patients is still unknown and deserves future investigation.

In our study, we found that all TB-DM people and a good proportion of TB-preDM patients were anemic. There is evidence supporting the idea that performance of HbA1c in detecting dysglycemia is affected by occurrence of anemia [35]. Herein, we analyzed the relationship between HbA1c and anemia and found no clear influence in the test performance to diagnose DM. Furthermore, our results demonstrated a low accuracy of the HbA1c vs. other tests to diagnose preDM, irrespective of the TB status. FPG was a better predictor of preDM than HbA1c in every condition tested. These observations suggest that FPG is indeed a good marker to be used for screening of DM or preDM in Peru, in addition to OGTT.

Our study has some limitations. OGTT was not tested on individuals who were recruited and a prior diagnosis of DM. This led to incomplete testing in all the study participants. In addition, we had study dropouts, which although were not very high, resulted in reduced number of individuals who were investigated in the latter study visits. Finally, the HbA1c levels could have been affected by the degree of anemia, as demonstrated before [36, 37]. Our study was not designed to directly test the effect of anemia on changes in HbA1c levels and thus further research is warranted to address this question. Regardless of such limitations, our study results clearly show the high prevalence and heterogeneity of DM and preDM in a Peruvian population, corroborating with observations reported from other countries such as India [38].

## Conclusions

The results presented here reveal a previously under notified high prevalence of DM and preDM in TB patients from Peru. Thus, screening strategies for both DM and preDM should be implemented in under routine program conditions for all patients. Using only HbA1c can lead to an error of sub-notification of preDM so the additional implementation of FPG and OGTT is needed in all cases where dysglycemia is suspected but not confirmed. Implementation of such screening tests in routine investigation at TB clinics could result in early detection of hyperglycemia and timely therapeutic interventions, which lead to improvements of clinical outcomes.

## Additional file

Additional file 1: **Table S1.** Sensitivity and specificity analysis of the FPG and HbA1c tests for detecting diabetes or prediabetes in the study population. (DOCX 19 kb)

## Data Availability

The datasets used and/or analysed during the current study available from the corresponding author on reasonable request.

## Abbreviations

ADA: American Diabetes Association
AFB: Acid-Fast Bacilli
ATT: Antitubercular treatment
BCG: Bacillus Calmette–Guérin
BMI: Body Mass Index
DM: Diabetes mellitus
FPG: Fasting plasma glucose
Hb: Hemoglobin
HbA1c: Glycated Hemoglobin
HHC: Household contact
IQR: Interquartile range
L-J: Löwenstein-Jensen
MDR: Multi Drug Resistant
Mtb: *Mycobacterium tuberculosis*
NTP: National TB Program
OGTT: Oral glucose tolerance test
preDM: Prediabetes
ROC: Receiver Operator Characteristic;
SEIS: Socios En Salud Informatic System
TB: Tuberculosis
WHO: World Health Organization
