# Supplementary material for: High prevalence and heterogeneity of Dysglycemia in patients with tuberculosis from Peru: a prospective cohort study
Source: BMC Infect Dis. 2019 Sep 11;19:799. doi: 10.1186/s12879-019-4416-2 (PMC6737721; doi:10.1186/s12879-019-4416-2)
Supplement: Supplementary file 1 — Table S1. Sensitivity and specificity analysis of the FPG and HbA1c tests for detecting diabetes or prediabetes in the study population. (DOCX 19 kb) [file 12879_2019_4416_MOESM1_ESM.docx]

**Supplemental Table 1.** Sensitivity and specificity analysis of the FPG and HbA1c tests for detecting diabetes or prediabetes in the study population.

| **Test** | **Study participants** | **Sensitivity (95%CI)** | **Specificity (95%CI)** | **Positive Predictive Value (95%CI)** | **Negative Predictive Value (95%CI)** | **p-value** |
| --- | --- | --- | --- | --- | --- | --- |
| **FPG** | **All individuals** |  |  |  |  |  |
|  | DM | 0.54 (0.40-0.68) | 0.55 (0.50-0.60) | 0.11 (0.08-0.16) | 0.92 (0.88-0.95) | 0.27 |
|  | PreDM | 0.75 (0.64-0.83) | 0.55 (0.50-0.60) | 0.25 (0.20-0.31) | 0.92 (0.88-0.94) | <0.0001 |
|  | **TB cases** |  |  |  |  |  |
|  | DM | 0.53 (0.36-0.70) | 0.54 (0.47-0.60) | 0.15 (0.09-0.23) | 0.88 (0.81-0.93) | 0.56 |
|  | PreDM | 0.71 (0.55-0.83) | 0.54 (0.47-0.60) | 0.23 (0.16-0.31) | 0.91 (0.84-0.95) | 0.01 |
|  | **HHC** |  |  |  |  |  |
|  | DM | 0.62 (0.36-0.82) | 0.56 (0.50-0.63) | 0.08 (0.04-0.15) | 0.96 (0.91-0.98) | 0.26 |
|  | PreDM | 0.78 (0.64-0.87) | 0.56 (0.50-0.63) | 0.27 (0.20-0.35) | 0.92 (0.87-0.96) | <0.0001 |
| **HbA1c** | **All individuals** |  |  |  |  |  |
|  | DM | 0.45 (0.31-0.60) | 0.45 (0.40-0.50) | 0.08 (0.05-0.12) | 0.89 (0.83-0.92) | 0.27 |
|  | PreDM | 0.25 (0.17-0.36) | 0.45 (0.40-0.50) | 0.08 (0.05-0.12) | 0.75 (0.69-0.80) | <0.0001 |
|  | **TB cases** |  |  |  |  |  |
|  | DM | 0.47 (0.30-0.64) | 0.47 (0.40-0.53) | 0.12 (0.07-0.18) | 0.85 (0.77-0.91) | 0.56 |
|  | PreDM | 0.29 (0.17-0.45) | 0.47 (0.40-0.53) | 0.09 (0.05-0.16) | 0.78 (0.69-0.84) | 0.01 |
|  | **HHC** |  |  |  |  |  |
|  | DM | 0.38 (0.18-0.64) | 0.44 (0.37-0.50) | 0.04 (0.02-0.09) | 0.92 (0.85-0.96) | 0.26 |
|  | PreDM | 0.22 (0.13-0.36) | 0.44 (0.37-0.50) | 0.08 (0.04-0.13) | 0.73 (0.65-0.80) | <0.0001 |

FPG: Fasting Plasma Glucose; HbA1c: Glycated Hemoglobin, 95%CI: 95% confidence interval. Data were analyzed using C-statistics.
